# Systematic review on the treatment of pentoxifylline in patients with non-alcoholic fatty liver disease

**DOI:** 10.1186/1476-511X-10-49

**Published:** 2011-04-08

**Authors:** Wenjun Li, Liu Zheng, Chunjun Sheng, Xiaoyun Cheng, Liu Qing, Shen Qu

**Affiliations:** 1 Department of Endocrinology, Shanghai Tenth People's Hospital, Tongji University School of Medicine, Shanghai 200072, China; 2 Shenzhen Bei Da Hospital, Shenzhen 518036, China; 3 Beijing You An Hospital, Beijing100005, China

## Abstract

**Background:**

As an anti-TNF agent that targets inflammatory process directly, Pentoxifylline has been investigated for treatment of NASH in individual studies and pilot trials for years. We summarized the available information and generating hypotheses for future research.

**Data Sources:**

Google, Cochrane, MEDLINE, and EMBASE and the *Chinese Biomedical *data bases for studies restricted to pentoxifylline treatment in humans with NAFLD in all languages until June 2010. Six studies (2 randomized, double-blind, placebo-controlled trials; 4 prospective cohort studies) extracted from 11604 references.

**Results:**

Pentoxifylline-treated patients showed a significant decrease AST (n = 37, *P *= 0.01) and ALT (n = 50, *P *= 0.03), but no significant effect on IL-6 (n = 36, *P *= 0.33) and TNF-α (n = 68, *P *= 0.26) compared with Placebo or UDCA-controlled groups. Improvement in one or more histological variables was reported in two trails, only 1 study showed a reduction in of one or two points in fibrosis stage.

**Limitations:**

The trails did not consistently report all of the outcomes of interest. Sample sizes (117 patients totally) were small and only 2 out of 6 studies had a randomized, controlled design.

**Conclusion:**

Pentoxifylline reduce AST and ALT levels and may improve liver histological scores in patients with NALFD/NASH, but did not appear to affect cytokines. Large, prospective, and well-designed randomized, controlled studies are needed to address this issue. Novel therapeutic targets for activation of inflammatory signaling pathways by fat also merit investigation.

## Introduction

Although, the pathogenesis of non-alcoholic steatohepatitis (NASH) is multifactorial [1-4], strong evidence of clinical observations [5,6]and animal models[7,8] indicated that inflammatory activation clearly plays a pivotal role in the disease progression. Chronic inflammation interplaying with increased oxidative stress, cytokine production, direct "lipotoxicity", and autoimmunity, is implicated in NAFLD pathophysiology by increasing NASH, fibrosis, and insulin resistance[9,10]. Patients with NASH have generally significantly higher levels of serum TNF-α and IL-6 than seen in patients with simple steatosis[11,12]. Furthermore, the expression of cytokines is higher in those patients with more severe NASH. Among the proinflammatory molecules, TNF-α has been proposed to be the key link between obesity and insulin resistance[13]. Thus, therapeutic strategies that directly target inflammatory pathway or secondarily alter the inflammatory process may influence both histological manifestations and systemic inflammatory state in patients with NASH.

There is currently no FDA approved available treatment for NASH. In recent years, the anti-TNF alpha agent pentoxifylline has been considered for treatment of NASH[14-16]. Pentoxifylline is a non-selective phosphodiesterase inhibitor reported to decrease TNF-α gene transcription as well as affecting multiple steps in the cytokine/chemokine pathway by direct or indirect inhibition of TNF-α[17-19]. It is necessary to examine the current state of limitation and beneficial effect of the treatment with pentoxifylline in patients with NAFLD or NASH and generating hypotheses for future research. However, we could not find any systematic reviews or meta-analyses addressing the issue. Therefore, the purpose of the research synthesis is to summarize the efficacy and safety of pentoxifylline for NASH in terms of plasma cytokines, aminotransferase, liver histopathology and adverse events.

## Methods

### Literature Sources and Search

**Figure 1:**
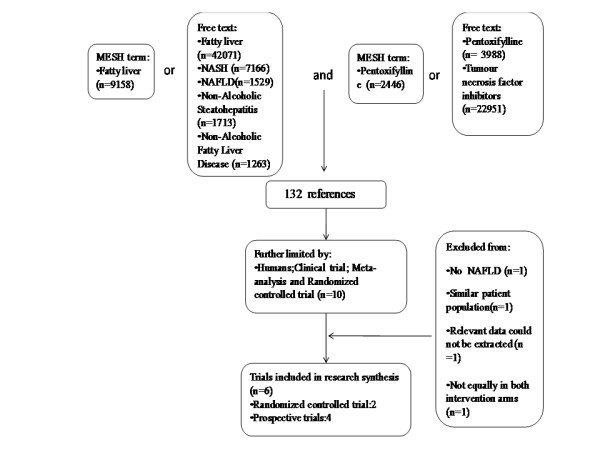
**Search strategy and results**. We use the Fatty liver as the MeSH term and list all known synonyms as free-text, 11604 references were searched totally. Combined search for all MeSH and free-text searches, 132 references were identified. Of these, we excluded 122, because they were animal experiments, nonclinical, or clearly irrelevant. Of the remaining 10 references, 4 were excluded because they did not meet our inclusion criteria. Finally, 6 references were identified (2 randomized trials and 4 prospective cohort studies) in the Figure 1 explained section.

We searched under Google, Cochrane, MEDLINE, and EMBASE and the Chinese Biomedical data bases for studies restricted to pentoxifylline treatment in humans with NAFLD/NASH in all languages until June 2010. Figure 1 shows our search strategy. We also searched the abstracts of Annual Meeting of the American and European Association for the Study of Liver in 2009 and 2010.

A separate search filtering for meta-analysis and clinical reviews yielded zero articles.

### Data Extraction and Outcome Definition

For each trial, the data was gathered on the following characteristics (Table 1.):

**Table 1:** Characteristics of 6 Trials on pentoxifylline in subjects with NAFLD

Source	Methodological quality	Diagnosis	Pentoxifylline/Control (numbers)	Interventions		Outcome
					
				Pentoxifylline	Control	
LeeYin-Mei, 2008[20]	Randomized and placebo-controled	NASH	11/9	Pentoxifylline (1200 mg/day for 12 weeks) plus low-calorie diet and daily exercise	Placebo plus low-calorie diet and daily exercise	AST, ALT, IL-6, TNF-a, adverse events
Buranawati W, 2007[21]	Randomized and placebo-controled	NASH	16/16	Pentoxifylline (1200 mg/day for 12 weeks) plus low-calorie diet	Placebo plus low-calorie diet	AST, ALT, TNF-a
Tuncer I, 2003[22]	Prospective cohort study with concurrent control	NAFLD	10/10	pentoxifylline (20 mg/kg/day for 24 weeks)	ursodeoxycholic acid	AST, ALT
Georgescu, EF 2007[23]	Prospective cohort study with concurrent control	NASH	13/13	Pentoxifylline (800 mg/day for 30 weeks)	Ursodeoxycholic acid	ALT, IL-6, TNF-a, Histology
Adams 2004[24]	Prospective cohort study with historical control	NASH	20	Pentoxifylline (1,600 mg/day) for 12 months		Adverse events
Satapathy SK 2007 [25]	Prospective cohort study with historical control	NASH	9	Pentoxifylline (1200 mg/day for 12 months)		Histology

1. NAFLD/NASH: diagnosed on persistently elevated aminotransferas > 1.5 times, plus liver biopsy and/or imaging techniques;

2. Type of studies: randomized and controlled trials, or prospective cohort studies with a control (concurrent or historical) group;

3. Pentoxifylline was administered at any dose, duration, and given singly or in combination versus placebo or other interventions. Co-interventions were allowed if used equally in both intervention arms of the trial.

Disagreements were resolved through consensus. Primary investigators were contacted if data were incomplete.

#### Primary outcomes

1. Mean change from baseline of ALT, AST levels and cytokines.

2. Histological response (number of patients with histological improvement/deterioration and changes in the degree of fatty liver infiltration, inflammation, and fibrosis) based on the Brunt system or its modifications[26,27].

#### Secondary outcomes

Safety and tolerability of pentoxifylline (any adverse events as reported in trials) in patients with NAFLD or NASH.

### Assessment of Methodological Quality and Statistical Analysis

The quality of the trials eligible for inclusion in the review were assessed by four (Wenjun Li, Liu zheng, Liu qing and Qu Shen) independent reviewers. Subsequently, other investigators and these four reviewers confirmed whether eligible studies met the inclusion criteria and independently assessed the accuracy of data extraction. If necessary, conflict was resolved by consensus of all investigators. The randomized, controlled trials were considered as high-quality evidence, prospective cohort studies with a concurrent control group as intermediate-quality evidence, and studies with a historical control group as low quality evidence. None of retrospective cohort studies was found.

Data was analyzed using the RevMan 5 provided by The Cochrane Collaboration. The results of the primary outcomes: mean change from baseline of ALT, AST levels and cytokines are presented as weighted mean differences (WMD) with 95% confidence intervals (CI). We used a fixed-effect model with the significant level set at P value = 0.05. Heterogeneity was explored by chi-squared test with significance set at P value ≤ 0.10 and the quantity of heterogeneity was measured by I^2^. Furthermore, a sensitivity analysis on the impact of the four excluded trials was performed in both the experimental and control study groups. For the secondary outcomes (safety and tolerability of Pentoxifylline) and histological response, we adopted available case analysis.

## Results

### Identification of Eligible Trials

Six studies met the specified criteria for assessment of ALT, AST and cytokines (table 1). Only two studies (Yin-Mei Lee and Buranawati W) are randomized double-blind, placebo-controlled trials[20,21]. Four prospective cohort studies with control groups includes two concurrent controls [22,23] and two historical controls[24,25]. In addition, two trails [24,25]with historical controls was not involved in the analysis of ALT, AST, and cytokines, but histology and adverse events information was extracted from them[24,25].

### Characteristics of Patients and Interventions

Six trials included 117 patients with biopsy proven NASH (n = 97) or NAFLD with high ALT and AST plus US or biopsy (n = 20). Combination of studies resulted in 48 total patients for the control group(25 for Placebo plus low-calorie diet or daily exercise, 23 for ursodeoxycholic acid alone)and 79 patients for the pentoxifylline group(27 pentoxifylline plus a low-calorie diet or daily exercise and 52 pentoxifylline alone). All trials used at a dose of 800 mg to 1600 mg per day for 3-6 months. Inclusion Criteria: Male and female patients ages 18 to 70 years; daily alcohol intake of < 20 g; appropriate exclusion of other liver diseases and positive testing for hepatitis B surface antigen, hepatitis C virus antibody, or RNA of hepatitis C virus and DNA of hepatitis B virus.

### Effects on Serum Aminotransferase

**Figure 2:**
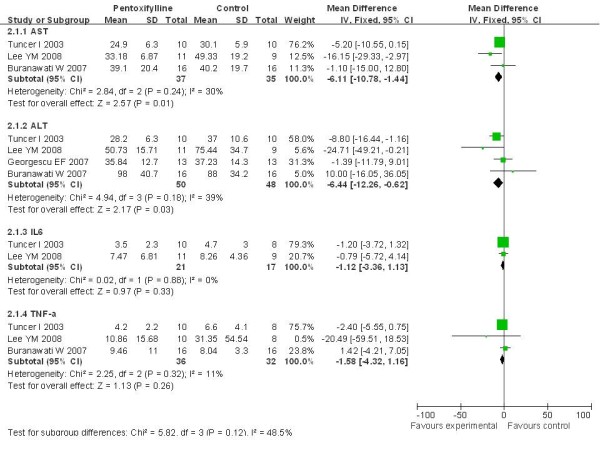
**Overall summary of meta-analyses results of treatments of pentoxifylline for NASH**. Pentoxifylline-treated patients showed a significant decrease AST (n = 37, *P *= 0.01) and ALT (n = 50, *P *= 0.03), but no significant effect on IL-6 (n = 36, *P *= 0.33) and TNF-α (n = 68, *P *= 0.26) compared with Placebo or UDCA-controlled groups.

Liver function response was assessed by serum activities of AST and ALT. AST activity was evaluated in all included trials, however, Placebo or UDCA-Controlled studies were reported only in three trials, including two randomized studies[20,21] and one prospective study [22]. Pentoxifylline-treated patients showed a significant decrease (P = 0.01) in AST activity (weighted mean differences (WMD) -6.11 IU/L, 95% CI -10.78 to -1.44) versus control-treated patients. Heterogeneity was high, but not statistically significant (I^2 ^= 30%, *P *= 0.24)(Figure 2).

ALT activity was evaluated in all four Placebo or UDCA-Controlled trials[20-23]. There were significant differences between pentoxifylline and control treatment (P = 0.03, WMD was -6.44 IU/L, 95% CI -12.26 to 0.62). Intertrial heterogeneity was not statistically significant (*P *= 0. 18) (Figure 2).

### Effects on Cytokines

IL-6 and TNF-α were able to be extracted from 2 Placebo [20,21] and 1 UDCA-controlled trials [22]. Meta-analyses of these data indicated that pentoxifylline had no significant effect on IL-6 (*P *= 0.33, WMD = -1.12, 95% CI: -3.39, -1.15) and TNF-α (*P *= 0.26, WMD = -1.58, 95% CI: -4.32, -1.16). In these analyses, intertrial heterogeneity was not statistically significant (*P *= 0.89 and *P *= 0.32, respectively) (Figure 2).

### Histological Response

Two trials [23,25] have assessed the histological response that evaluated the scores of steatosis, inflammation/necrosis, and fibrosis before and after treatment. After 12 months of therapy with pentoxifylline, significant overall improvement of liver histology was reported in Satapathy SK's study. Sixty seven percent of patients had a decrease in overall grading based on Brunt's staging (*P *= 0.009). Fifty-five percent of the patients had a reduction in their steatosis and lobular inflammation (RR, 0.06: 95% CI, 0.00 to 1.43). Four out of the six patients with baseline fibrosis had a reduction in their fibrosis stage (3 had one stage reduction and 1 patient had a two stage reduction), and another two patients remained stable on follow-up biopsy. In Georgescu's study, each histological variable before and after treatment was significantly diminished from 2.231 to 1.462 for steatosis score, from 1.692 to 1.231 for the lobular inflammation score, from 1.308 to 1.154 for hepatocyte ballooning score and from 1.308 to 1.077 for fibrosis score None of these scores changed significantly in the placebo and UDCA group.

### Adverse events

Among the six trials, two [20,24] provided information on adverse events. In Adams's trial [24], nine of 20 patients withdrew from the study, primarily because of nausea, but no serious adverse events occurred. One explanation for the significant drop out rate in the study is probably due to a higher dose of pentoxifylline used (1,600 mg/day). Pentoxifylline was well tolerated at a dose of 1,200 mg/day for six months and no adverse events and none was lost to follow-up in the trial [20]

## Discussion

Pentoxifylline has been safely used for the treatment of other medical conditions such as alcohol related liver disease. Pentoxifylline-treated patients have a significant mortality and hepatorenal syndrome improvements [28]. Individual studies and pilot trials of pentoxifylline in nonalcoholic steatohepatitis have suggested that anti-inflammatory therapy had effectively biochemical improvement and cytokine-mediated systemic inflammation amelioration (i.e., reduced plasma TNF-α and IL-6 levels). These results serve as "proof of concept" that pentoxifylline has efficacy in patients with nonalcoholic steatohepatitis, but the overall benefits of this approach have not been fully assessed quantitatively.

Improved liver aminotransferase levels are consistent evidence from all trails in the research synthesis. Pentoxifylline reduced significantly ALT and AST levels in patients with NASH compared to the placebo or UDCA groups. Increased liver enzymes are usually thought to be a consequence of liver injury in NAFLD, and improvement in liver enzymes can possibly be used as predictors of NAFLD improvement[29]. However, some studies have shown that a significant proportion of patients with normal ALT also had steatohepatitis and fibrosis on liver biopsy[30-32], indicating that aminotransferase per se cannot be used as a surrogate marker to assess the significant potential in therapies in patients with NASH.

Histological response was assessed only by Satapathy SK and Georgescu EF[25,23]. Both studies evaluated the scores of steatosis, inflammation/necrosis, and fibrosis before and after treatment. Improvement in one or more histological variables was noticed only in pentoxifylline subgroup, but not in the UDCA subgroup in the Georgescu EF's study. A significant reduction in steatosis, ballooning and lobular inflammation was also observed in the Satapathy SK's study. It was interestingly to be noted that pentoxifylline had a reduced baseline fibrosis stage in four out of the six patients, including 3 patients had one stage and a single patient had a two stage reduction [25]. Because fibrosis may progress in up to one third of patients with nonalcoholic steatohepatitis, regardless of whether hepatic aminotransferases improves or worsens, the antifibrotic effects of pentoxifylline should surely be an important issue. The overall methodological quality of the studies included in this issue was relatively weak and some bias may exist. Both are prospective studies with a historical [25] or a concurrent [23] control group, thus the true benefits may not be as extreme as reported here.

As an anti-TNF agent, pentoxifylline may be valuable in amelioration of cytokine-mediated systemic inflammation in patients with NASH. However, there was no significant difference in the reduction of TNF-α and IL-6 between the pentoxifylline and the control groups[20-22]. Because of a lack of comprehensive data in most studies, we could not reliably assess these factors. An protocol of ongoing trial by Mary ER et al[33] is available online, which assess the effect of pentoxifylline on adipocyte-derived cytokines, leptin and adiponectin, and its effect on free fatty acid levels in patients with NASH, but we was not able to find the final report.

Despite the limitations of the studies included in this review, it seems to suggest that pentoxifylline therapy had a beneficial effect on liver aminotransferase levels and histology improvements (reductions in steatosis, inflammation, and ballooning necrosis). Large, prospective, and well-designed randomized, controlled studies are needed to address this issue. It will probably be important to design specific anti-inflammatory approaches for different types and stages of NAFLD subgroups[34]. In addition, novel 'anti-inflammatory' targets evolving from basic research, such as activation of inflammatory signaling pathways by fat, in particular IKK and JNK, or Foxo1, also merit investigation[35].

## Competing interests

The authors declare that they have no competing interests.

## Authors' contributions

WL, ZL, QL and QS as the independent reviewers carried out data collection and analysis; QL and QS participated in the design of the study and performed the statistical analysis. WL, CS and XC confirmed whether eligible studies met the inclusion criteria and independently assessed the accuracy of data extraction. All authors read and approved the final manuscript.

## References

[B1] Reddy JK Rao MS Lipid Metabolism and Liver Inflammation. II. Fatty liver disease and fatty acid oxidation Am J Physiol Gastrointest Liver Physiol 2006 290 G852 G858 10.1152/ajpgi.00521.2005 16603729

[B2] Schonfeld G Yue P Lin X Chen Z Fatty liver and insulin resistance: not always linked Trans Am Clin Climatol Assoc 2008 119 217 23 discussion 223-4 18596860 PMC2442699

[B3] Samuel VT Liu ZX Qu X Elder BD Bilz S Befroy D Romanelli AJ Shulman GI Mechanism of hepatic insulin resistance in non-alcoholic fatty liver disease Biol Chem 2004 279 32345 53 10.1074/jbc.M313478200 15166226

[B4] Postic C Girard J Contribution of de novo fatty acid synthesis to hepatic steatosis and insulin resistance: lessons from genetically engineered mice J Clin Invest 2008 118 829 38 10.1172/JCI34275 18317565 PMC2254980

[B5] Dowman JK Tomlinson JW Newsome PN Pathogenesis of non-alcoholic fatty liver disease QJM 2010 103 71 83 10.1093/qjmed/hcp158 19914930 PMC2810391

[B6] Maher JJ Leon P Ryan JC Beyond insulin resistance: Innate immunity in nonalcoholic steatohepatitis Hepatology 2008 48 670 8 10.1002/hep.22399 18666225 PMC3592568

[B7] Feldstein AE Canbay A Angulo P Taniai M Burgart LJ Lindor KD Gores GJ Hepatocyte apoptosis and fas expression are prominent features of human nonalcoholic steatohepatitis Gastroenterology 2003 125 437 443 10.1016/S0016-5085(03)00907-7 12891546

[B8] Schattenberg JM Singh R Wang Y Lefkowitch JH Rigoli RM Scherer PE Czaja MJ JNK1 but not JNK2 promotes the development of steatohepatitis in mice Hepatology 2006 43 163 172 10.1002/hep.20999 16374858

[B9] Abiru S Migita K Maeda Y Daikoku M Ito M Ohata K Nagaoka S Matsumoto T Takii Y Kusumoto K Nakamura M Komori A Yano K Yatsuhashi H Eguchi K Ishibashi H Serum cytokine and soluble cytokine receptor levels in patients with non-alcoholic steatohepatitis Liver Int 2006 26 39 45 10.1111/j.1478-3231.2005.01191.x 16420507

[B10] Bahcecioglu IH Yalniz M Ataseven H Ilhan N Ozercan IH Seckin D Sahin K Levels of serum hyaluronic acid, TNF-alpha and IL-8 in patients with nonalcoholic steatohepatitis Hepatogastroenterology 2005 52 1549 53 16201116

[B11] Perseghin G Petersen K Shulman GI Cellular mechanism of insulin resistance: potential links with inflammation Int J Obes Relat Metab Disord 2003 27 Suppl 3 S6 S11 10.1038/sj.ijo.0802491 14704736

[B12] Shoelson SE Lee J Goldfine AB Inflammation and insulin resistance J Clin Invest 2006 116 1793 1801 10.1172/JCI29069 16823477 PMC1483173

[B13] Choi S Diehl AM Role of inflammation in nonalcoholic steatohepatitis Curr Opin Gastroenterol 2005 21 702 7 10.1097/01.mog.0000182863.96421.47 16220049

[B14] Perseghin G Petersen K Shulman GI Cellular mechanism of insulin resistance: potential links with inflammation Int J Obes Relat Metab Disord 2003 27 Suppl 3 S6 S11 10.1038/sj.ijo.0802491 14704736

[B15] Lee JY Hwang DH The modulation of inflammatory gene expression by lipids: mediation through Toll-like receptors Mol Cell 2006 21 174 185 16682810

[B16] Lorenzo M Fernández-Veledo S Vila-Bedmar R Garcia-Guerra L De Alvaro C Nieto-Vazquez I Insulin resistance induced by tumor necrosis factor-alpha in myocytes and brown adipocytes J Anim Sci 2008 86 14 Suppl E94 104 17940160 10.2527/jas.2007-0462

[B17] Diehl AM Tumor necrosis factor and its potential role in insulin resistance and nonalcoholic fatty liver disease Clin Liver Dis 2004 8 619 638 10.1016/j.cld.2004.04.012 15331067

[B18] Desmouliere A Xu G Costa AM Yousef IM Gabbiani G Tuchweber B Effect of pentoxifylline on early proliferation and phenotypic modulation of fibrogenic cells in two rat models of liver fibrosis and on cultured hepatic stellate cells J Hepatol 1999 30 621 631 10.1016/S0168-8278(99)80192-5 10207803

[B19] Bahra PS Rainger GE Wautier JL Nash GB Effects of pentoxifylline on the different steps during adhesion and transendothelial migration of flowing neutrophils Cell Biochem Funct 2001 19 249 257 10.1002/cbf.922 11746205

[B20] Lee YM Sutedja DS Wai CT Dan YY Aung MO Zhou L Cheng CL Wee A Lim SG A randomized controlled pilot study of Pentoxifylline in patients with non-alcoholic steatohepatitis (NASH) Hepatol Int 2008 2 196 201 10.1007/s12072-008-9058-1 19669304 PMC2716847

[B21] Buranawati W Thoun-U-Thaisri Pramoolsinsup C Wisedopas N Atamasirikul K Udomsubpayakul U Pentoxifylline for Treatment of Nonalcoholic Fatty Liver Disease (NAFLD): A Randomized, Placebo-Controlled Study Thai J Gastroenterol 2007 8 57 63

[B22] Tuncer İ Uygan İ Dülger H Türkdoğan K Şekeroğlu MR Kösem M The comparative effects of pentoxifylline and ursodeoxycholic acid on IL-1β, Il-6, Il-8 and TNF-α levels in nonalcoholic fatty liver Eastyern Journal of Medicine 2003 8 27 32

[B23] Georgescu EF Georgescu M Therapeutic options in non-alcoholic steatohepatitis (NASH). Are all agents alike? Results of a preliminary study J Gastrointestin Liver Dis 2007 16 39 46 17410287

[B24] Adams LA Zein CO Angulo P Lindor KD A pilot trial of pentoxifylline in nonalcoholic steatohepatitis Am J Gastroenterol 2004 99 2365 2368 10.1111/j.1572-0241.2004.40064.x 15571584

[B25] Satapathy SK Sakhuja P Malhotra V Sharma BC Sarin SK Beneficial effects of pentoxifylline on hepatic steatosis, fibrosis and necroinflammation in patients with non-alcoholic steatohepatitis J Gastroenterol Hepatol 2007 22 5 634 8 17444848 10.1111/j.1440-1746.2006.04756.x

[B26] Brunt EM Nonalcoholic steatohepatitis: pathologic features and differential diagnosis Semin Diagn Pathol 2005 22 330 338 10.1053/j.semdp.2006.04.002 16939061

[B27] Yeh MM Brunt EM Pathology of nonalcoholic fatty liver disease Am J Clin Pathol 2007 128 837 847 10.1309/RTPM1PY6YGBL2G2R 17951208

[B28] Akriviadis E Botla R Briggs W Han S Reynolds T Shakil O Pentoxifylline improves short-term survival in severe acute alcoholic hepatitis: a double-blind, placebo-controlled trial Gastroenterology 2000 119 1637 48 10.1053/gast.2000.20189 11113085

[B29] Adams LA Angulo P Lindor KD Nonalcoholic fatty liver disease CMAJ 2005 172 899 905 10.1503/cmaj.045232 15795412 PMC554876

[B30] Wong VW Chan HL Hui AY Chan KF Liew CT Chan FK Sung JJ Clinical and histological features of non-alcoholic fatty liver disease in Hong Kong Chinese Aliment Pharmacol Ther 2004 20 45 49 10.1111/j.1365-2036.2004.02012.x 15225170

[B31] Loguercio C De Girolamo V de Sio I Tuccillo C Ascione A Baldi F Budillon G Cimino L Di Carlo A Di Marino MP Morisco F Picciotto F Terracciano L Vecchione R Verde V Del Vecchio Blanco C Non-alcoholic fatty liver disease in an area of southern Italy: main clinical, histological, and pathophysiological aspects J Hepatol 2001 35 568 74 10.1016/S0168-8278(01)00192-1 11690701

[B32] Dixon JB Bhathal PS O'Brien PE Nonalcoholic fatty liver disease: predictors of nonalcoholic steatohepatitis and liver fibrosis in the severely obese Gastroenterology 2001 121 91 100 10.1053/gast.2001.25540 11438497

[B33] Rinella Mary E Pentoxifylline/Nonalcoholic Steatohepatitis (NASH) Study: The Effect of Pentoxifylline on NASH Clinical trials.gov 2005

[B34] Matteoni CA Younossi ZM Gramlich T Boparai N Liu YC McCullough AJ Nonalcoholic fatty liver disease: a spectrum of clinical and pathological severity Gastroenterology 1999 116 1413 9 10.1016/S0016-5085(99)70506-8 10348825

[B35] Liu Qing Bengmark Stig Qu Shen The role of hepatic fat accumulation in pathogenesis of non-alcoholic fatty liver disease (NAFLD) Lipids in Health and Disease 2010 9 42 10.1186/1476-511X-9-42 20426802 PMC2873482

